# A Hybrid Lightweight System for Early Attack Detection in the IoMT Fog

**DOI:** 10.3390/s21248289

**Published:** 2021-12-11

**Authors:** Shilan S. Hameed, Ali Selamat, Liza Abdul Latiff, Shukor A. Razak, Ondrej Krejcar, Hamido Fujita, Mohammad Nazir Ahmad Sharif, Sigeru Omatu

**Affiliations:** 1 Malaysia-Japan International Institute of Technology (MJIIT), University Teknologi Malaysia, Kuala Lumpur 54100, Malaysia; hameed.s@graduate.utm.my; 2 Directorate of Information Technology, Koya University, Koya 44023, Iraq; 3 School of Computing, Faculty of Engineering, Universiti Teknologi Malaysia, Skudai 81310, Malaysia; shukorar@utm.my; 4 Media and Games Center of Excellence (MagicX), Universiti Teknologi Malaysia, Skudai 81310, Malaysia; 5 Center for Basic and Applied Research, Faculty of Informatics and Management, University of Hradec Kralove, Rokitanskeho 62, 50003 Hradec Kralove, Czech Republic; ondrej.krejcar@uhk.cz; 6 Razak Faculty of Technology and Informatics, Universiti Teknologi Malaysia, Kuala Lumpur 54100, Malaysia; liza.kl@utm.my; 7 i-SOMET Incorporated Association, Morioka 020-0104, Japan; 8 Regional Research Center, Iwate Prefectural University, Takizawa 020-0693, Japan; 9 Institute of IR4.0, Universiti Kebangsaan Malaysia, Bangi 43600, Malaysia; mnazir@ukm.edu.my; 10 Graduate School, Hiroshima University, Kagamiyama, Higashihiroshima 739-8511, Japan; omtsgr@gmail.com

**Keywords:** IoMT, IoT, hybrid attack detection, incremental learning, machine learning, sensor’s data, NetFlow data, NIDS, HIDS, fog computing

## Abstract

Cyber-attack detection via on-gadget embedded models and cloud systems are widely used for the Internet of Medical Things (IoMT). The former has a limited computation ability, whereas the latter has a long detection time. Fog-based attack detection is alternatively used to overcome these problems. However, the current fog-based systems cannot handle the ever-increasing IoMT’s big data. Moreover, they are not lightweight and are designed for network attack detection only. In this work, a hybrid (for host and network) lightweight system is proposed for early attack detection in the IoMT fog. In an adaptive online setting, six different incremental classifiers were implemented, namely a novel Weighted Hoeffding Tree Ensemble (WHTE), Incremental K-Nearest Neighbors (IKNN), Incremental Naïve Bayes (INB), Hoeffding Tree Majority Class (HTMC), Hoeffding Tree Naïve Bayes (HTNB), and Hoeffding Tree Naïve Bayes Adaptive (HTNBA). The system was benchmarked with seven heterogeneous sensors and a NetFlow data infected with nine types of recent attack. The results showed that the proposed system worked well on the lightweight fog devices with ~100% accuracy, a low detection time, and a low memory usage of less than 6 MiB. The single-criteria comparative analysis showed that the WHTE ensemble was more accurate and was less sensitive to the concept drift.

## 1. Introduction

Smart health systems such as the Internet of Medical Things (IoMT) and Medical Cyber–Physical Systems (MCPSs) are a subset of the Internet of Things (IoTs) [1]. They are gaining popularity via simple fitness gadgets connecting athletes to their smartphone devices and cloud services [2]. IoMT is a broad technology, incorporating various products and platforms, including implanted devices, eldercare wearables for monitoring [3], internet-connected clinical equipment, and remote-surgery hospital rooms [4]. Medical gadgets and devices are classified into four groups, based on their closeness to the patient’s body and their safety levels [5], as illustrated in Table 1.

Medical biosensor devices monitor biological vitals and send enormous volumes of biodata in real-time [6]. Later, these data are aggregated and pre-processed in personal databases, such as cellphones and laptops, or other devices such as routers and access points at the fog layer [7]. Subsequently, the pre-processed data are landed in massive hospital servers, which can act as the cloud databases. Generally, machine learning technologies are employed at the cloud or hospital server for attack detection. Fog computing is a new technology invented to overcome the cloud’s delay, centralization, and privacy concerns. As a result, some cloud computing duties have been moved to the closest area to the smart devices. This new concept seems to be very compatible with the medical IoT. It speeds up the patient data analytics at the fog layer and protects data from privacy vulnerabilities, because the information does not need to be transferred to the cloud [6,8,9]. It is undeniable that these innovations contribute to the enhancement of care systems, thereby providing a healthier life, and an increased life expectancy [1,10]. Nevertheless, most clinical and consumer medical equipment are vulnerable to numerous cyber-attacks [4,11,12]. The causes of these vulnerabilities include hardware and software defects [13], such as the lack of security measures in small personal devices and installing outdated operating systems and susceptible apps on clinical devices [12,14]. Moreover, software updates are not always possible and applicable for use by healthcare staff, whereas hackers are always devising new and sophisticated cyber-attacks [15]. Furthermore, some recent applications, such as virtual assistants on medical devices, which are not secure, will put the devices at risk [16]. Hence, securing the whole system of the IoMT system requires all the stakeholders to secure the main three layers of the system. There are different possible attacks on the IoMT, targeting different system layers. The most recurrent and known attacks against the device and network layer are given in Table 2. Consequently, developing defensive systems such as threat intelligence and intrusion detection systems that employ machine learning technologies are of great importance.

Various strategies in machine learning (ML) have been applied to detect attacks [25,31]. Nevertheless, the attack detection in the IoMT devices is challenging because ML-based heavy techniques put too much burden on these devices [4]. Therefore, in some studies, the use of external gadgets is considered as a solution. For instance, in study [21], the authors installed their model on an external device to detect radio frequency signals received and sent by medical devices using a multi-layer threat detection, by which all attacks can be detected. The authors of [32] proposed a statistical-based solution, using an external hardware device to detect code injection attacks. It was seen that the system did not impose extra overhead on medical devices. Moreover, edge devices such as Raspberry Pi3 and deep learning (DL) were used for detecting false data injections in the implantable brain devices [33]. In another study, multi-layer perceptron (MLP) and field-programmable gate array (FPGA) chips were used for attack detection in insulin pumps [34]. Furthermore, authors in [35] used principal component analysis (PCA) and a correlation coefficient (CC) feature selection to detect false sensor readings using mobile computing. On the other hand, the researchers of [36] used mobile computing and Markov model-based techniques to inject false data, using ECG data. Nevertheless, the device’s performance was limited, which made them unusable for the ever-growing sensor data unless a lightweight incremental method was used. Therefore, cloud-based attack detection was alternatively proposed to detect attacks against medical devices. For instance, researchers proposed a cloud-based system to detect and prevent resource depletion attacks targeting an implantable cardioverter-defibrillator [37], in which the system was divided into six sub-layers. One layer was devoted to the ML technique using one-class SVM; however, the experiments on the actual healthcare data showed that one class SVM could not achieve a super detection rate, due to insufficient attack samples.

Nevertheless, attack detection at the cloud is characterized by delay, and it is also not distributed, making it less effective for distributed and time-sensitive systems such as the medical IoT. Fog-based attack detection is more compatible with the IoMT system. This is because it does not utilize the medical devices themselves for heavy computation. Furthermore, it is much closer to the medical devices, leading to an instant response when there is an attack in a distributed manner. Therefore, a fog-based attack detection that is distributed and closer to the edge network can solve the above limitations. Related to the fog-based attack detection in other domains, a group of researchers focused on implementing deep learning on fog devices for the DDoS attack detection [38,39,40]. However, their proposed systems cannot be installed on lightweight devices. In another study, researchers developed a lightweight model using MLP and network traffic data in the fog layer [41], showing the effectiveness of the fog-based system for network attack detection. However, the proposed system was not hybrid. The authors of [42] proposed a fog-based data intrusion detection system (DataIDS) to detect false data injection in medical devices. An attack tree was also considered as a threat model of Fog/IoT scenarios with heterogeneous devices. Their fog-based model was able to reduce the overhead on medical devices. However, the model was not validated to be lightweight. De Donno et al. [43] proposed an anti-malware solution that used fog computing to safeguard IoT devices from DDoS Mirai attacks. They specifically examined the system’s architecture and development, such as security considerations, components of the system, and their interaction. However, the proposed approach was a prototype, and it has not been validated using ML techniques.

In the medical IoT, fog-based attack detection has been employed in only a handful of research [44,45,46]. Alrashdi et al. [44] proposed a fog computing-based intrusion detection system using an online sequential extreme learning machine (EOS-ELM) for a smart medical system. They proved that their distributed fog-based architecture was better than cloud-computing-based architecture, as it had a lower detection time and a higher detection rate. Moreover, the authors of [45] built an intrusion detection system for network attack detection in smart medical systems, utilizing fog–cloud architecture. Their experiment used an ensemble of Decision Tree, Naïve Bayes, and Random Forest to form XGBoost, whereas cloud computing and fog computing were used for the training and testing processes. Moreover, an ensemble incremental learning approach was also employed for the network intrusion detection of fog devices in the medical IoT, using the NSL-KDD dataset [46]. As such, the current work was motivated by the outcome of an extensive literature review, which led to the identification of the following problems.

Firstly, previous studies have focused on either a host-based or network-based attack detection system. However, having a hybrid detection system is essential for the simultaneous detection of malicious sensor and network data. Second, the previous models are not lightweight, making them incompatible with fog devices. This is because the sensor and network data of the IoMT system have increased with time (yielding big data), and hence the fog devices are unable to hold them.

Consequently, it would be beneficial to use data incrementally and adaptively to avoid a retraining process. Therefore, the main objective of this work is to build a hybrid lightweight and adaptive fog-based attack detection system for medical devices and their networks (hybrid system). The system comprises five single online incremental learning methods and a novel ensemble technique. This is the first work presented that employs adaptive incremental learning for hybrid attack detection in the IoMT fog, to the best of our knowledge. In this way, the model can detect early attacks, due to its incremental featured nature. Furthermore, it uses multi-sensor and NetFlow data, which is more compatible with medical systems as it does not use payload analysis, thereby avoiding privacy violation. Hence, the contributions of this work can be summarized as follows:

- A fog-computing architecture for the IoMT system is proposed, representing moderate to large healthcare organizations.
- We propose a hybrid (for host and network) lightweight fog-based multi-attack detection system with early detection capability, due to adaptive learning techniques. These methods are novel Weighted Hoeffding Tree Ensemble (WHTE); Incremental Naïve Bayes (INB); Hoeffding Tree Majority Class (HTMC); Hoeffding Tree Naïve Bayes (HTNB), and Hoeffding Tree Naïve Bayes Adaptive (HTNBA). The model is unique because it was performed by an adaptive incremental setting, which does not impose any overhead on the devices. Consequently, the proposed model is suitable for real-world circumstances, with ever-increasing IoT-infected sensors and NetFlow data with multiple attacks arriving at the fog devices.

Therefore, the advantage of this approach is two-fold: first, the attacks can be identified as soon as they arrive at the sensors and the network (hybrid and early detection); second, the detection model uses a short memory (lightweight). Additionally, the detection system is adaptive to the new incoming sensor and network traffic since it does not forget the model’s current detection ability. Instead, it updates the model based on the new incoming data.

The remainder of the paper is organized as follows: the proposed systems, their methods, and architecture are given in Section 2, while in Section 3, the results and details of the findings are discussed. Finally, the conclusions along with limitations and future works are drawn.

## 2. Materials and Methods

### 2.1. Proposed Fog-Based Hybrid Attack Detection System

**Figure 1:**
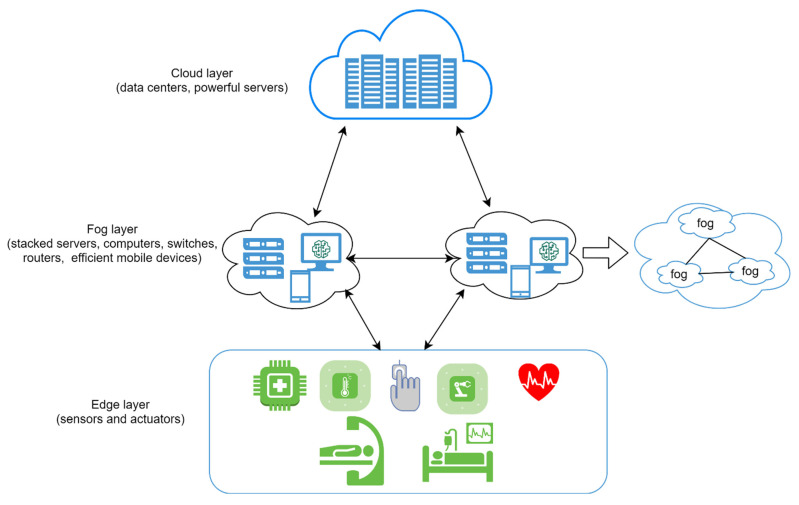
The proposed architecture of fog computing-based medical IoT is inspired by the IEEE standard [48].

We followed the IEEE standard for the IoMT fog-based system in the proposed architecture, as illustrated in Figure 1. Any devices with computing and storage capability such as gateways, embedded devices, and personal servers can be used as a fog node in this architecture [47]. Furthermore, this functions as the first security point for tiny medical devices with no security measures [48]. One can see from Figure 1 that the proposed fog architecture consists of one head cluster, followed by a lower layer of more than one sub-head cluster, until it reaches the edge devices. Additionally, the analytical ability decreases from the top to the bottom of the architecture, with increased raw data.

**Figure 2:**
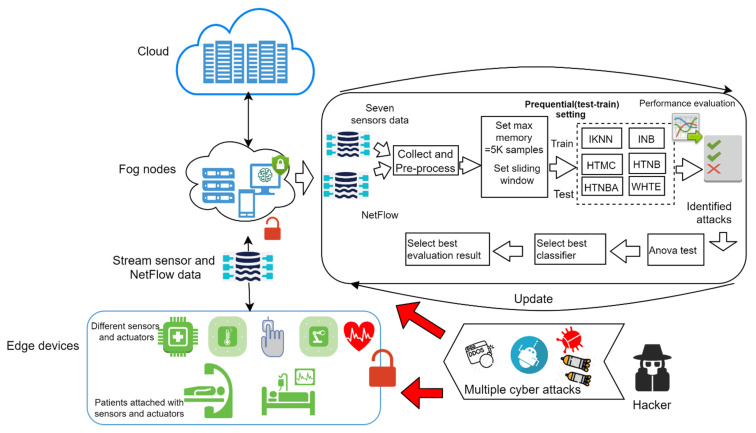
The framework of the proposed hybrid attack detection system.

The main framework of the system, combining the architecture and the overall flowchart steps of the detection system, is illustrated in Figure 2. As can be seen in Figure 2, the red unlocked icon indicates missing security protocols at the edge devices and network connections among edge devices and fog nodes. Hence, the devices at the edge layer and the network connection between them and the fog layer are vulnerable to multiple cyber-attacks (details are given in Section 2.2), due to the lack of security measures at that layer. Therefore, the hackers can easily break the system and flood it with attacks. Furthermore, the sensor data which is transmitted by the network to the fog layer in seconds is infected by such attacks. As such, the steps of the proposed framework are summarized as follows:

- Collecting and extracting the sensor and network data at the edge of the IoMT system. The extracted datasets are explained in detail in Section 2.2.
- Preprocessing the collected data to remove noises, followed by utilizing a sliding window to set the ML classifiers. By this, the memory is updated whenever a new set of samples arrives, and set to the sliding window. The sliding window was set to one thousand records and the memory frequency was set to five thousand records at a time.

**Figure 3:**
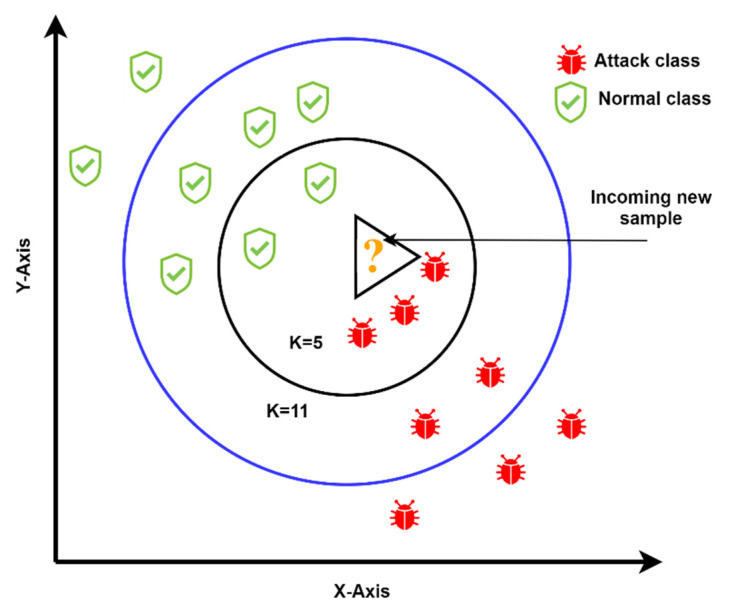
The IKNN method predicts new data as an attack or normal, based on different K-values and distance equations.

**Figure 4:**
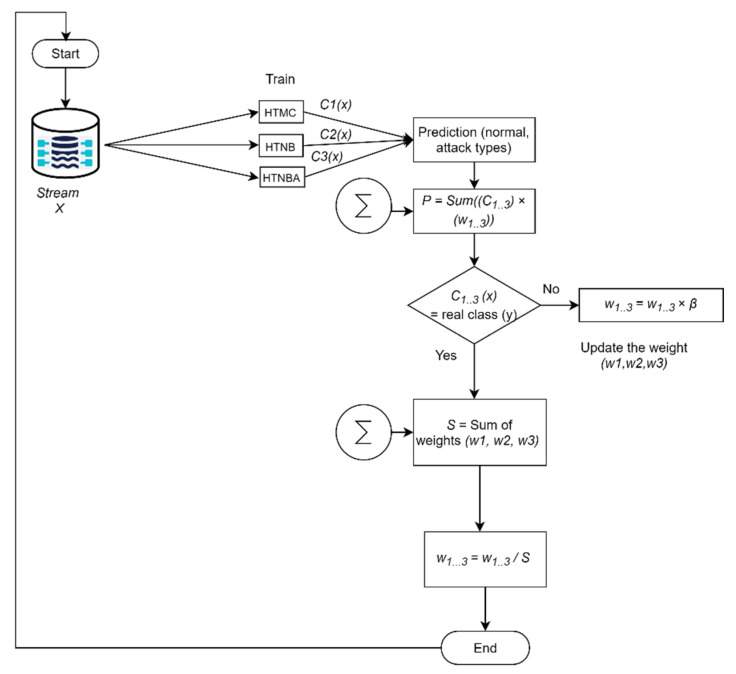
The flowchart of the proposed ensemble WHTE method.

Thus, when many instances are used, they will be discarded from memory and replaced by the following five thousand records. The arc with the update label in Figure 2 indicates restarting the process continuously, once data arrives. Then, a combination of single incremental classifiers with the ensemble WHTE is used for the purpose of learning and prediction, in which a prequential learning approach is employed to include every single sample for training and testing. The employed machine learning classifiers, their parameter tuning, and further details, are given below:

1. Incremental K-Nearest Neighbour (IKNN): This is an incremental modified version of the original K-Nearest Neighbour, a supervised machine learning technique for classification and regression problems. It uses the concept of similarities (also known as distance), such as finding the distance between two points or two objects on a surface. The KNN technique assumes that the samples close to each other belong to the same class. The parameters to be fed to this technique are the K-value, and the number of nearest neighbours that decide whether an instance belongs to those nearest neighbors’ class. Another parameter is the mathematical method for calculating the distance. In this work, the value of N was set to 10, and the mathematical approach was a linear model. Figure 3 illustrates how IKNN predicts new samples when they arrive at the system. If we take a smaller circle, in which the K value is five, the labels of the five nearest samples are examined. Then, based on the most repeatable class, it will decide the class of the new sample. Therefore, when K is five in the smaller circle, the incoming sample will be considered a threat, because the nearest samples are at least three. However, if the K value is 11, as shown in the larger circle, the incoming new sample will be considered normal, because there are six near-normal samples.
2. Incremental Naïve Bayes: Naïve Bayesian classification is a common classification system used to analyze large sizes of data. In this work, Naïve Bayes incremental learning is used to handle massive datasets on resource-limited fog devices. The method’s success is that it can be supplemented with updated data without storing the entire dataset in the memory. Because of its incremental feature, the algorithm is well suited for detecting and classifying attacks quickly and accurately [49]. The basic formula of Naïve Bayes is as follows:
  (1) $$P(y|X)=\frac{P(X|y)P\left(y\right)}{P\left(X\right)}$$
  where, y is the class, which takes values considering the type of attacks and normal class, and X is the list of features, in which X can be a list of components identified as $X=\left\{x_{1},x_{2},x_{3},x_{4}\ldots x_{n}\right\}$. If this list is replaced by the variable X, the equation can be updated as follows:
  (2) $$P(y|x_{1},\ldots ,x_{n})=\frac{P\left(x_{1}|y\right)\ldots \ldots P(x_{n}|y)P\left(y\right)}{P\left(x_{1}\right)\ldots P\left(x_{n}\right)}$$
  Since the denominator of the above equation remains static, we can remove it and proportionally inject it into the equation as follows:
  (3) $$P\left(y|x_{1},\ldots ,x_{n}\right)\propto P\left(y\right)\prod_{i=1}^{n}P\left(x_{i}|y\right)$$
  This is for binary classes; however, we have eight classes in our datasets. Hence, the class with the highest probability or likelihood should be chosen by:
  (4) $$y={\mathrm{argmax}}_{y}P\left(y\right)\prod_{i=1}^{n}P\left(x_{i}|y\right)$$
  From Equation (4), we can predict the class value for a list of features.
3. Hoeffding Tree-based classifiers: Different forms of the original Hoeffding Tree (HT) are implemented in this study. The details of these classifiers are explained below:
  - Hoeffding Tree-Based Majority Class (HTMC): Conventional tree-based classifiers have a significantly restricted number of training instances, because they presume that the real data should be saved in memory at once [50]. Hoeffding Tree (HT) is an efficient and straightforward tree-based classifier, designed to stream big data. This tree-based classifier assumes a constant distribution of data. This method works based on Hoeffding Bound’s theory. The Hoeffding Tree has a theoretically appealing feature that other incremental tree-based learners do not have. It provides a solid efficiency guarantee. HT is initially designed for big data streams. Hoeffding Tree-based Majority Class (HTMC) is a form of HT that uses the majority class technique to decide about the classes of the tree branches [51].
  - Hoeffding Tree Naïve Bayes: There are some efforts to improve the accuracy of this classifier by using other techniques at the HT leaves. Using Naïve Bayes classifiers rather than the majority class classifier improves the HT classification performance. Most of the class approach utilizes information on classes patterns, only to categorize a test sample without searching for feature values. This only makes use of a small portion of the information given, and it is a rough estimation of the pattern of instances. However, Naïve Bayes considers both the prior distributions of the classes and the likelihoods of a feature, given the class. This allows for far better utilization of the available information [51]. The sample is traversed from the root to the leaf, to classify new samples. It uses the splitting test and feature values to establish a correct path. When the sample reaches any leaf, it will classify the sample. Here, the classifier will be a Naïve Bayes classifier. It uses information gained during the classification process without adding overhead [51].
  - Hoeffding Tree Naïve Bayes Adaptive (HTNBA): At the presence of noise in cyber-attack infected datasets, Naïve Bayes at leaves may not tolerate well, and its performance is not better than the HTMC. Therefore, a hybrid of majority class and Naïve Bayes is proposed to overcome both techniques’ shortcomings, called Hoeffding Tree Naïve Bayes Adaptive (HTNBA) [52]. The HTNBA method uses Naïve Bayes Adaptive to classify the instances based on their discriminative features. It also uses the Gain value to decide the class of the incoming instances (Holmes et al., 2005). After predicting the new instances by both Naïve Bayes and the majority class, the hybrid method chooses one of them based on their prediction value.
4. Weighted Majority Hoeffding Tree Ensemble (WHTE): single classifiers which were explained previously may not work similarly well on different types of input data. Therefore, their ensemble can be used to maximize their performance and minimize their weaknesses. Since sensor and network data change in their features and sample statistics, applying one type of classifier may not always bring the best result. Therefore, we propose a new ensemble approach, combining a group of single classifiers; specifically, the different types of Hoeffding Tree classifiers (HTMC, HTNB, HTNBA). The ensemble approach uses the weighted majority approach, which initially considers all the classifier’s decisions [53]. However, it will punish a classifier once they make a wrong decision, by not considering their decisions to be as important as they were [54]. All experts (classifiers) will have a vote, and initially these will be equally weighted. Then, if one of those experts (classifiers) makes a mistake, their votes will be cut down in proportional value, and weighting will be used for the overall performance. Then, the final performance is optimal because the mistakes made by the entire algorithm will be close to the constant mistake made by the best technique. When the expert makes a mistake in the original weighted majority algorithm, the weighted value will be multiplied by ½. Hence, the error bound equation is as follows:
  (5) M≤2.41(m+logN)
  where m is the number of mistakes made by the best classifier, M is the number of errors made by the ensemble, and N is the number of classifiers in the pool.
  One can see that the minimum constant factor (coefficient of the right-hand side of Equation (5)) is greater than two. However, this can be reduced by performing the randomized form of weighted majority algorithm in which Beta (β) is used as a penalty value to minimize the constant value to be close to one. Therefore, in our WHTE algorithm, the error will be counted as follows:
  (6) $$M\leq \frac{m\ln\left(\frac{1}{\beta }\right)+\ln N}{1-\beta }$$
  In our study, we have set the value of β to be 0.5. Hence, the value of M for each iteration or a sample at a time will be counted by:
  (7) M≤1.39m+2lnN
  A simplified flowchart of our ensemble method is presented in Figure 4, whereas the pseudo code of the entire system is shown in Algorithm 1.

**Algorithm 1.** Hybrid Lightweight Attack Detection System
**Input** $S_{i}$ as a set of single sensor datasets, N as NetFlow dataset, $C_{j}$ as set of single incremental classifiers (INB, IKNN, HTMC, HTNB, HTNBA), and W as ensemble WHTE**Output** $P_{k}$ set of performance metrics (average accuracy, average memory, average CPU time, kappa statistic, precision, recall, concept drift sensitivity) **Begin** At fog devices close to the edge in framework 1 Collect infected sensor data S in $S_{i}$ Collect infected NetFlow N **for**
N and each S in $S_{i}$ **while** there is data record in S and N   **Do**      Pre-process the data     Set the sliding window to 1K records     Set the maximum memory to 5K records     Set the initial parameters of each C classifier in $C_{i}$      Set the parameters of each C in W     **for** each record in *S* and *N*       **Do**        Prequential train and test using W and each C $\mathrm{in}\;C_{i}$
        Compute each P in $P_{k}$
        **return** average $P_{k}$        Update parameters of W and each C in $C_{i}$   **end for**    **end while**      **for each**
C in $C_{i}$ and W       **for each** P in $P_{k}$       **Compute** one-way ANOVA          Assume that all population means are equal         Initialise *p* value         **if** (*p* < 0.05).         **then** reject         **else** accept         **return** statistical metrics         **end if**
      **end for**     **end for**    **end for**  **End**

### 2.2. Sensor and Network Datasets for IoMT Fog

**Figure 5:**
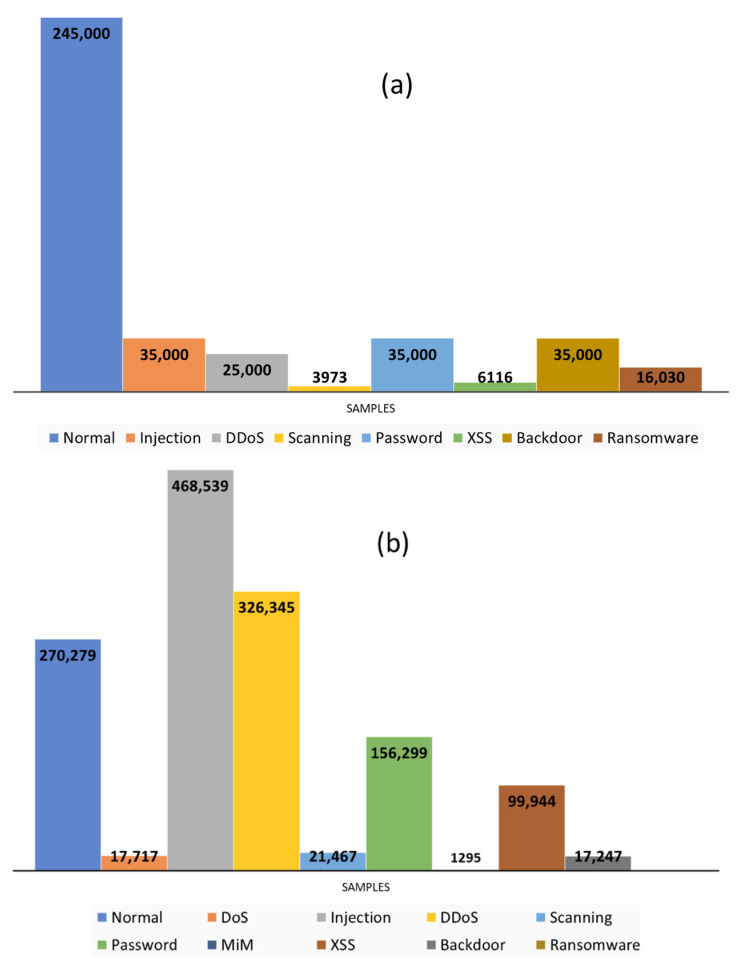
Number of attack samples in the sensor datasets (**a**) and NetFlow dataset (**b**).

In this study, we used seven sensors and telemetry data infected with multiple attacks for the host attack detection, whereas for the network detection, NetFlow data of the same fog-oriented framework was used. The datasets were created in a real environment of an IoT network, such as the fog-based architecture presented in Section 2.1. Therefore, the dataset represents the heterogeneous nature of the IoT system. Both sensor and network data were extracted from the same fog-based architecture while infected by the recent attacks. The details of the extracted datasets are as follows:

1. ToN-IoT: The first form of the dataset was a collection of sensors datasets gathered from the seven different sensors. It held data of about seven IoT cyber-attacks with legitimate device data. Each dataset had different features on the devices. The total samples of all datasets were 401,119 (fridge: 59,944, Garage door: 59,587, GPS: 58,960, Modbus: 51,106, Motion light: 59,488, Thermostat: 52,774, Weather: 59,260). The attacks in this dataset were (backdoor, distributed denial of service (DDoS), injection, password, ransomware, scanning, and cross-site scripting (XSS)) [55]. Figure 5a shows the number of attack samples among all the sensor datasets.
2. NetFlow_ToN-IoT: The second form of dataset was the network traffic of the IoT system, in which network traffic packets were transformed to NetFlow files [56]. NetFlow format network traffic was less heavy than its payloads (packets) because it used the traffic features, rather than the content of the packets. This dataset consisted of 14 features and 1,379,274 samples. This big data included all the attacks which existed in its sensor telemetry datasets, such as injection, DDoS, scanning, password, XSS, backdoor, and ransomware, with another two attacks of denial of service (DoS), and man in the middle (MiM), which mainly target the network layer. Additionally, the attacks at NetFlow had a different number of samples, compared with those in the sensor dataset. Figure 5b shows the number of attack samples in the NetFlow dataset.

### 2.3. Evaluation Metrics

- Average accuracy: This is the average of all the sliding windows’ accuracies at the end of the analysis. In incremental learning, accuracy is not calculated in this way in batch learning, because there is a high possibility for the samples to fall within a single class in one sliding window.

(8) $$\mathrm{Average}\;\mathrm{accuracy}=\frac{\sum_{i}acc}{N}$$

In which acc is the accuracy for the samples in each i sliding window over N (the total number of the sliding windows), whereas acc can be defined by:

(9) $$acc=\frac{Cc}{M}$$

where Cc is the correctly classified instance, and M is the total number of samples at that time.

- Average time (s): The average CPU time taken by the incremental learning method for each sliding window dataset’s training and evaluation process.
- Precision: Is the ratio of true positive over predicted positive samples.
- Recall: This is the ratio of true positive over total positives in the dataset.
- Kappa Static: It measures how well the samples identified by the classifier resembled the data labeled as ground truth, while adjusting for the performance of a randomized classifier as evaluated by anticipated accuracy. It will add more information to the accuracy of a model, especially when the incoming samples are more skewed (imbalanced).
- ANOVA test: To evaluate the significant differences among the performances of the used classifiers, one-way ANOVA is used.

### 2.4. Experimental Environment

The proposed system was designed for an IoMT framework, as presented in Section 2.1 The detection system can be installed on any device or agent that is close to the edge of the network. Hence, the used devices in our experiment were set to be at the lower tier of the fog layer. An example of that is a PC with Corei5 5200U (4 CPUs ≈ 2.2 GHz, and 8 GB memory). Furthermore, we set the window size to 5000 samples, which was compatible with lightweight devices. The incremental data collection and analysis simulation was performed using well-known machine learning tools, such as Python and the associated incremental learning package scikit-multiflow, combined with a java-based MOA package.

## 3. Results and Discussion

### 3.1. Host-Based Attack Detection

The detection system needed to simultaneously use all the IoT sensor readings to capture any malicious readings for the host-based attack detection. Hence, the readings of the infected IoT sensors were used in the detection system, and their results were analyzed and compared accordingly. It was seen that the proposed model was able to achieve a high average accuracy close to 100% for all the datasets, indicating that the system could detect all the attacks at the same time. It can be noticed from Table 3 that all the classifiers performed well, where the bolded accuracies are the highest ones. However, the ensemble WHTE was generally better than the other methods. Additionally, the INB and HTNBA performed better than the IKNN, HTNB, and HTMC.

**Figure 6:**
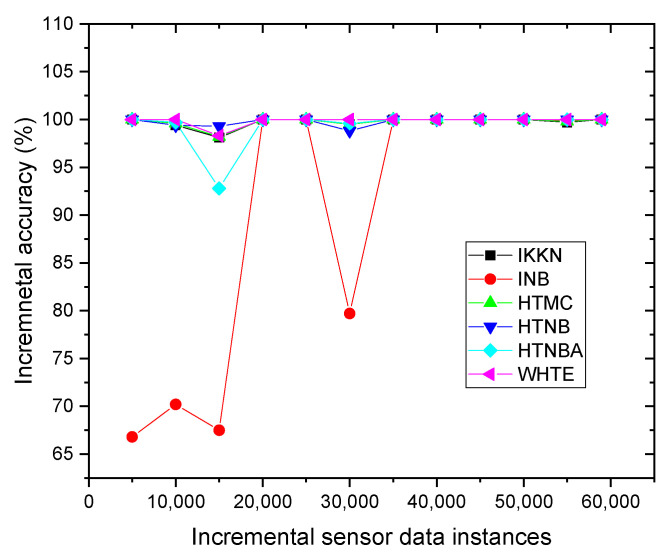
The incremental accuracy of the applied methods for each subset of data samples per slide window for the GPS-tracker sensor dataset.

Since the proposed system used incremental learning instead of batch learning, it is informative to show how each technique in the system reacted with increasing instances. This helped in identifying the accuracy for each sliding window. Therefore, the GPS-Tacker sensor dataset was chosen and analyzed to observe the range of the total accuracy for each classifier, and to notice how they were updated by adding up the new samples in the sliding windows. It was seen from Figure 6 that the INB and HTNBA performance fluctuated during different sample frequencies, showing that these two techniques were not stable, and were affected by concept drift in the data. Nevertheless, the other techniques, especially the WHTE ensemble, remained stable throughout the sample frequencies.

**Figure 7:**
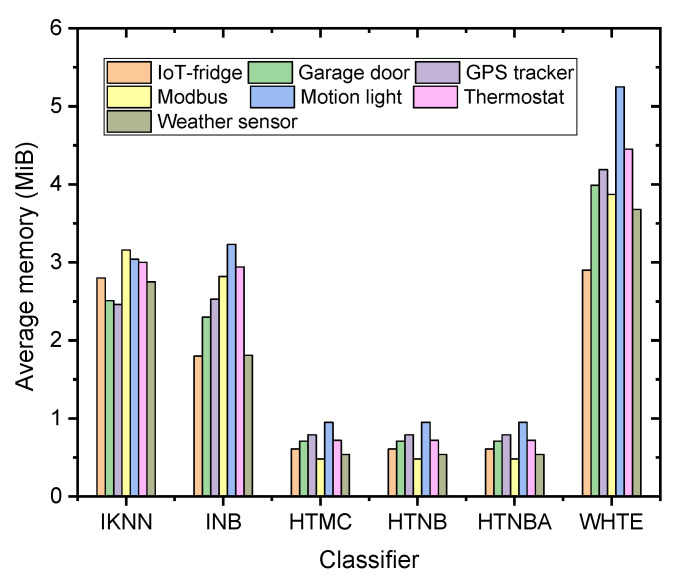
The comparison of the proposed methods in terms of average memory usage among all the datasets.

Compared with the models reported in the literature, the system proposed herein showed a lightweight nature. It was seen from the results of the CPU time complexity shown in Figure 7 that the average memory usage was located between 0.48 and 0.95 MiB for all the datasets using the HTMC, HTNB, and HTNBA methods, whereas it was between 2.46 and 3.16 MiB, and between 1.80 and 3.16 MiB for the IKNN, and INB methods, respectively. This memory usage was seen to be slightly larger for the WHTE algorithm, (2.9 to 5.25 MiB) because it was an ensemble of three techniques. Nevertheless, the maximum memory usage was less than 6 MiB, making it suitable for the lightweight nature of the fog devices. This ensures that the system can be installed on a mobile phone or a raspberry Pi device without adding overhead on the device.

**Figure 8:**
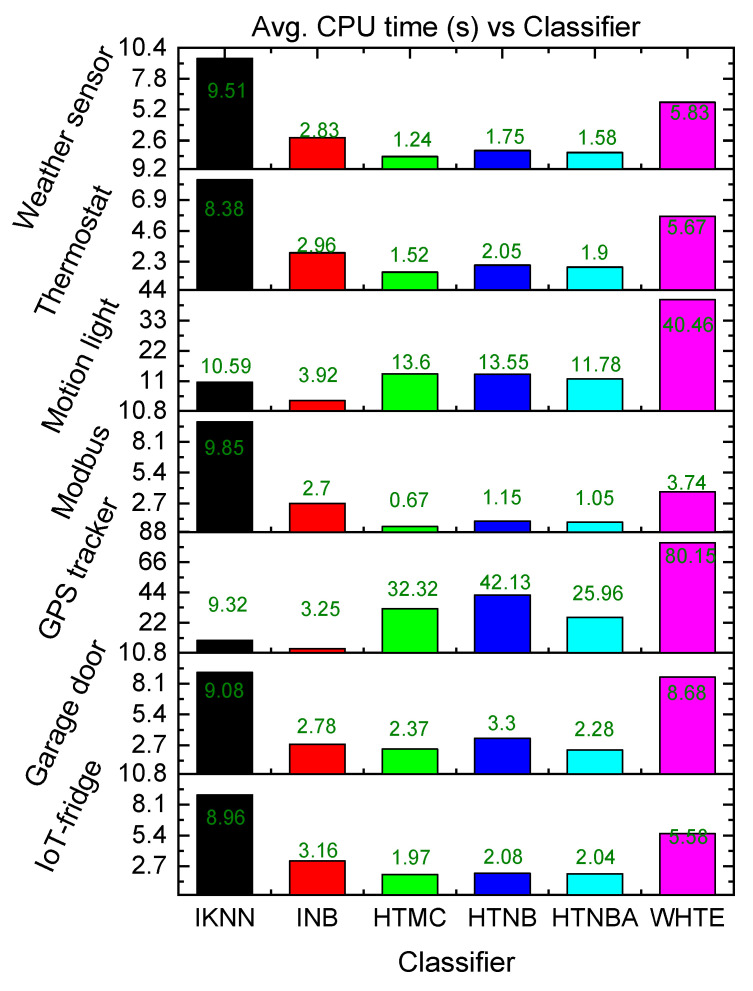
The comparison of the proposed classifiers in terms of average CPU time among all the datasets.

In addition to the lightweight feature of the proposed system, the system needs to rapidly detect the attacks in an early phase. Hence, the techniques were evaluated using the CPU time in terms of time complexity. It was seen that due to the adaptive sliding window, the processing time of the methods was significantly low, as shown in Figure 8. It is noteworthy that the average CPU time was found to be between 1.05 and 11 s. This was from 8.38 to 25.96 s, 0.67 to 32.32 s, 1.15 to 42.11 s, 2.70 to 3.90 s and 3.74 to 80.15 s for the HTNBA, IKNN, HTMC, HTNB, INB, and WHTE, respectively, among all the datasets. Therefore, the HTMC method recorded the lowest single CPU time. However, the INB method showed the lowest range of CPU time. Nevertheless, taking the method time into account, the detection time of all attacks was within a few seconds of their first appearance in the IoMT system, as shown in Table 3.

**Figure 9:**
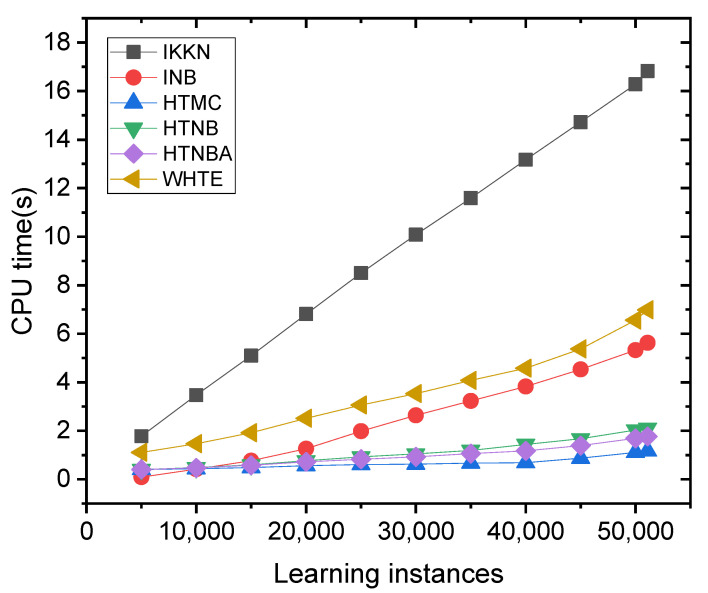
The comparison of the CPU time for all the methods applied on the dataset of the Modbus sensor.

To further study the proposed technique tolerance to the increased sample size, one can investigate the CPU time values. It is well known that increasing the number of instances acts upon increasing the CPU time. We chose the Modbus sensor dataset for this illustration, because all the methods were similarly accurate when applied to that dataset. One can see from Figure 9 that the computation time of the methods increased when the Modbus sensor learning instances arrived incrementally. This is more pronounced for the IKKN method than for the other methods. This can be related to the fact that the IKNN method becomes slower when new data samples are received [57].

To identify a significant difference among the performance of the different classifiers, we have performed a detailed statistical analysis. The single-factor variance analysis (ANOVA) was used to investigate the statistically significant difference between the utilized classifiers in terms of accuracy, time complexity, and memory usage. In this analysis, the null hypothesis assumes that all population means are equal; the alternative hypothesis is at least one mean difference. Therefore, the null hypothesis is rejected at the probability of less than 5% (*p* < 0.05). Table 4 shows the statistical results obtained when accuracy was considered the independent variable among the classifiers used. It was found that the average accuracy of the classifiers over the seven datasets was trivially changed, with the highest accuracy being for the WHTE method. It is noteworthy that the statistical result led to the calculation of a *p*-value of 0.10. Therefore, the null hypothesis was accepted, and hence there was no statistically significant difference between the algorithm’s performance in terms of accuracy. However, the proposed WHTE outperformed the other techniques when the overall accuracy was not considered statistically.

Table 5 shows the statistical results when computation time was the independent variable among the used classifiers. It was seen that each algorithm consumed different times to carry out the computation on the other datasets. Concludingly, the classifiers did not significantly differ in their time to perform the computational process. It is worth mentioning that the mean square (MS) of the variances within the classifiers was higher than that between the classifiers. One can observe that the *p*-value was 0.30, which was higher than 0.05.

Table 6 tabulates the calculated statistical results when the classifiers used memory usage as the independent variable. It was found that there was a relatively significant difference in the average value of memory usage between the classifiers. However, this difference was highly related to the IKNN, INB, and WHTE classifiers, compared with the other classifiers. Additionally, the variances in memory within the classifiers were low compared with that obtained between the classifiers. The HTMC, HTNB, HTNBA classifiers took less memory to perform computational tasks than the IKNN, INB, and WHTE ones. Consequently, the very small *p*-value of 2.5 × 10^−19^ rejects the null hypothesis and supposes a statistically significant difference between the classifiers in memory usage. The above significance analysis showed that the proposed system could be confirmed as a lightweight detection system for the host and sensor devices with a high detection rate.

### 3.2. Network-Based Attack Detection

After analyzing the sensor datasets, the NetFlow format of the network traffic of the testbed system was collected and analyzed, as was explained in the methodology section. The same techniques were applied to the NetFlow dataset, and results were obtained accordingly. It was seen that the proposed methods achieved high accuracy with low CPU time and memory usage. As shown in Table 7, the average accuracy of the proposed model was 100% for the ensemble WHTE. Additionally, the HTMC and HTNBA methods recorded the second-highest accuracy of 99.01%. The average memory usage was 0.37 MiB for the WHTE method and 1.15 MiB for the IKNN, whereas it was 0.08 MiB for the HTNBA, HTNB, and HTMC methods. This concluded that the network analysis of the attacks took less than 2 MiB, making it compatible with very lightweight devices. On the other hand, the average CPU time that was consumed for detecting all the attacks was only 12.89 s for the WHTE method, 5.02 s for the HTNBA method, and 3.90 s for the HTNBA method. However, the time complexity was high for the IKNN method.

It is known that network traffic data contains higher samples of normal traffic than attacks, which was the case for our datasets. Besides, in the incremental data analysis, there is always a high possibility that the data at any sliding window is skewed towards one of the classes. We also need to observe how the models precisely detect the attacks, by taking the precision and recall values. From Table 8, one can see that for normal traffic data the precision was more than the recall, whereas the attack precision was lower than the recall for the INB and HTNB. This indicated that most classifiers presented a higher detection rate for the normal rather than the attack samples. Noticeably, the ensemble WHTE showed a balanced recall and precision for both classes.

**Figure 10:**
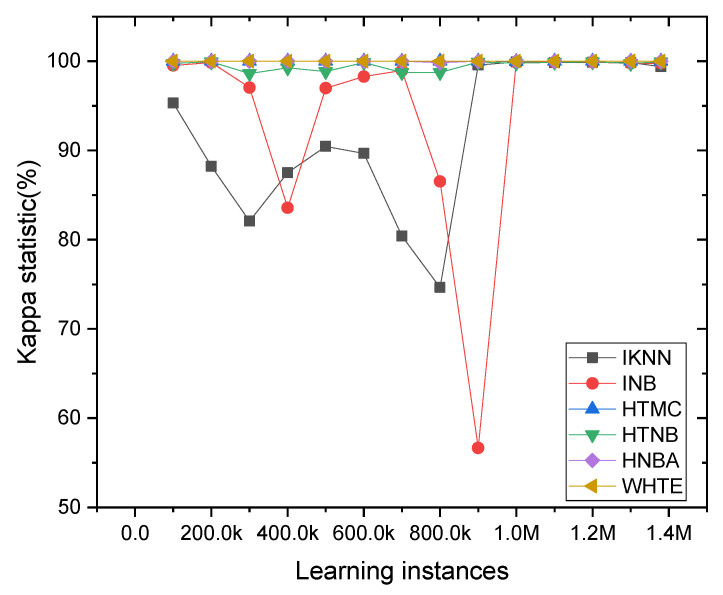
The classifier’s kappa statistic was compared per 5K sliding window samples in the NetFlow-ToNIoT dataset. For clear visualization, the kappa value was averaged for every 100K samples.

The accuracy value can be more viable when it matches the kappa statistic in these cases. Therefore, further analysis has been done using each classifier’s incremental kappa statistic values. It can be seen from Figure 10 that the ensemble WHTE method had the highest and most stable kappa value, throughout all the sample frequencies. Additionally, the HTMC and HTNBA models achieved a more stable kappa value among the other techniques.

**Figure 11:**
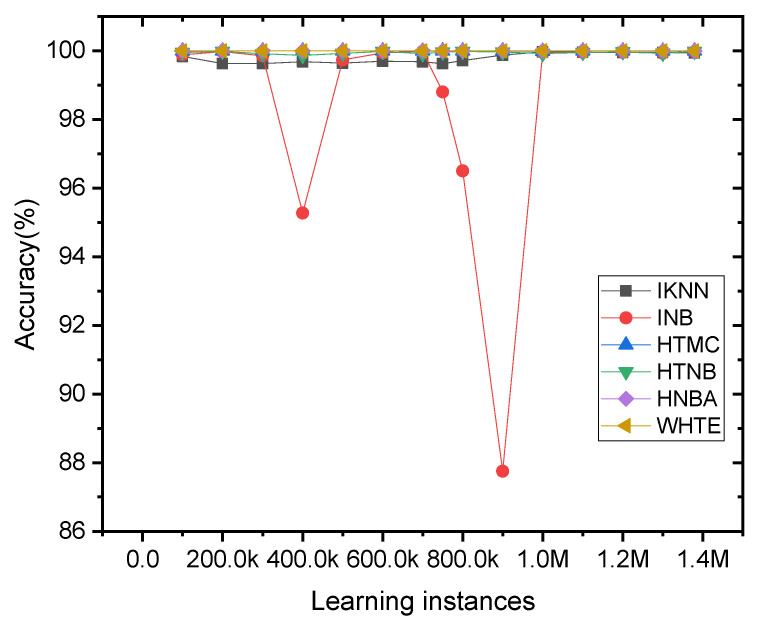
The incremental accuracy of the applied methods per 5K sliding window samples for the NetFlow-ToNIoT dataset. For clear visualization, the accuracy was averaged for every 100K samples.

To show how each classifier performed on the dataset when they arrived in an incremental fashion, a figure of the online learning techniques was conceptualized when the network traffic data was loaded to the system. One can observe from Figure 11 that the INB method faced a fluctuation in its accuracy, where an apparent variation can be seen in its accuracy spectra against different instances. Comparably, the other classifiers did not show significant sensitivity to the change in the dataset samples in the current form. This is because the ripples in the figure were suppressed due to the high variance in INB accuracy.

**Figure 12:**
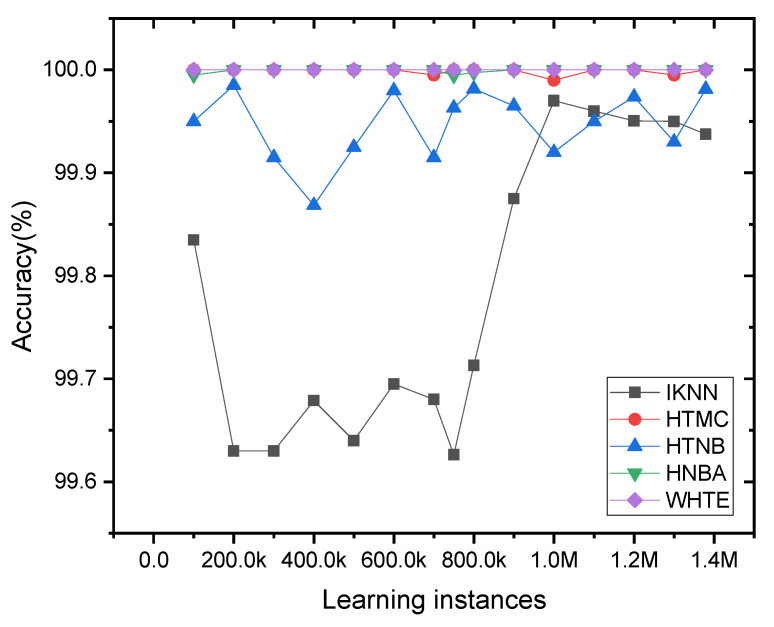
The incremental accuracy of the applied methods, except INB, per 5K sliding window samples for the NetFlow-ToNIoT dataset. For clear visualization, the accuracy was averaged for every 100K samples.

Therefore, to see the sensitivity of the other classifiers to the data variance (concept drift), a new plot was produced without the presence of the INB result, as shown in Figure 12. One can see that the HTNB and IKNN were slightly more sensitive to the concept drift than the other classifiers. The WHTE method attained the most stable performance with 100% accuracy for all the sample frequencies. Additionally, the HTMC and HTNBA showed a stable accuracy with less fluctuation than the other techniques. Hence, the WHTE, HTMC and HTNBA were the most reliable and durable classifiers in terms of sensitivity to the concept drift for the NetFlow-ToNIoT dataset.

**Figure 13:**
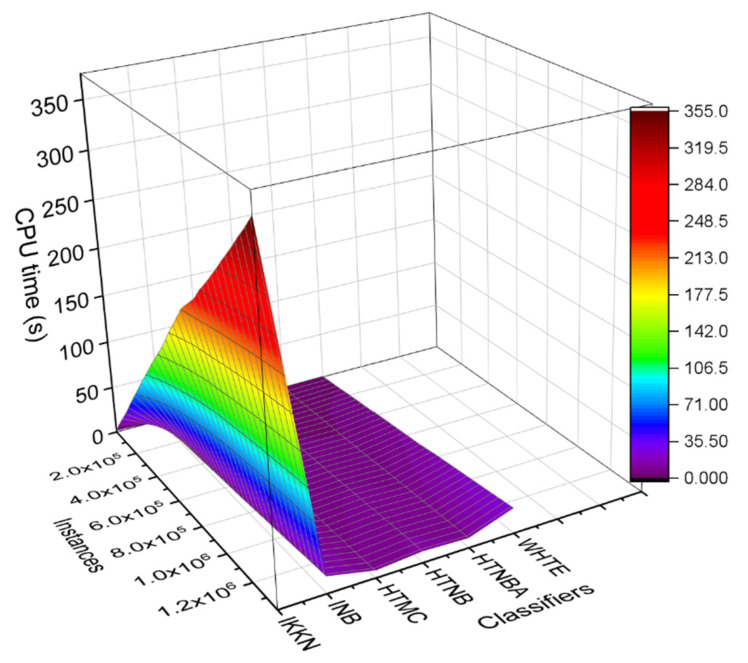
The 3D colormap surface compares the classifiers based on the CPU time for each subset of data samples per slide window in the NetFlow-ToNIoT dataset.

Further comparison was performed among the utilized techniques by using the NetFlow dataset. We had previously shown that the classifier’s CPU time was varied when the data was increased by time (see Figure 8). The same comparison was made for the NetFlow dataset utilizing a 3D colourmap surface, as shown in Figure 13. One can notice that the IKNN classifier was significantly affected by the increasing number of the instances in the dataset, where the surface colour is extended from light purple to dark brown, representing a higher slope of the relation, and hence a more significant increment rate. However, the other classifiers’ CPU time was not affected much by the increasing instances. More specifically, the CPU time was increased linearly with the increase in the sample load. However, the increment rate was more significant for the IKNN classifier than the other classifiers. It was seen from Figure 13 that the CPU time in the case of using the HTMC classifier was less affected by the increased instances. This is because the HTMC uses few mathematical operations. Noticeably, in the low range of the incremented samples, the CPU times for the HTMC and HTNBA were almost similarly increased. However, in the high range of the incremented samples, the CPU consumption for the HTNBA was larger than that of the HTMC method. Furthermore, the CPU time for the WHTE and INB methods was increased by adding the samples in each window.

Since the proposed work is a fog-based framework that uses incremental and ensemble adaptive learning for the host and network early attack detection, it is not common to compare every single result with the previous works that used batch learning. However, some generalized criteria can be used to compare the system to the related studies presented in Section 1. Table 8 shows a comparison of our achieved results with those reported in the literature. It was found that the accuracy (100%) and overall performance of our proposed model outperformed those reported in the literature. The proposed model was lightweight, considering the complexity of memory usage and CPU time approaches. Furthermore, as the device performance in the current study was considerably lower than those of the previous studies, the proposed model could be more compatible with less efficient devices at the fog layer, as shown in Table 8.

Additionally, the proposed model was designed for hybrid (network-based and host-based) multi-attack detection. On the contrary, the previous studies were designed for network-based attack detection only. Furthermore, our lightweight model’s computation did not affect the performance of the devices when it was installed as a host-based attack detector. When the model was used for attack detection at the networks of such devices, it did not impose any overhead on the bandwidth and communication links between the devices. Moreover, the designed model could handle the ever-increasing IoT data of sensors and networks.

It is worth mentioning that this study provides a comprehensive comparative analysis for the applied methods in terms of complexity and performance metrics, followed by statistical investigations. The results of this study were also compared with those of the previous works, based on extra factors which are shown in Table 8, where our proposed model outperformed the earlier works, thereby providing an efficient and lightweight fog model at the edge-fog layer. The model used incremental online learning, using six different classifiers, and can be used for hybrid multi-attack detection in IoMT devices and networks.

## 4. Limitations and Future Work

The proposed system was designed for multiple attack detection in sensors and network data, using lightweight fog devices. Since the system uses incremental learning, it may be affected by the concept drift, especially when the data has many features, and the features are constantly changed. Therefore, when the IoMT fog-based architecture has efficient fog devices, the proposed attack detection system could use batch learning to improve accuracy. Furthermore, in fog computing, collaborative attack detection is more compatible with its distributed nature. Therefore, a collaborative distributed learning could be considered in the future.

## 5. Conclusions

A new hybrid lightweight fog-based attack detection system was successfully established and proposed for IoMT devices and networks. The intelligent model comprised six incremental classifiers, including a novel ensemble incremental method called WHTE. Results on the recent fog-based sensor and network datasets showed that the system had achieved an accuracy of ~100% and low CPU time. Furthermore, the usage of memory was less than 6 MiB. The single-criteria comparative analysis showed that the WHTE ensemble was more accurate and less sensitive to concept drift issues. The proposed hybrid attack detection system can be installed on lightweight devices across the edge-fog layer without any overhead on the performance of the devices. This can be attributed to its adaptive incremental nature. Consequently, the proposed model outperformed the previously reported methods, in terms of performance and complexity.

## Figures and Tables

**Table 1:** Types of Medical IoT devices with their descriptions and examples.

Type	Description	Examples
Implantable 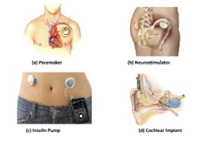	They are implanted in human organs.	Hip implants, cardiac pacemakers, implanted insulin pumps, and hearing implanted devices.
Wearable 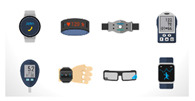	Humans wear these devices.	Smartwatch, fitness devices, etc.
Ambient 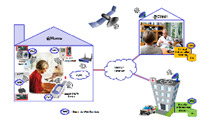	These devices are for monitoring human behaviors.	Telemetry devices for patient and remote elderly monitoring.
Stationary 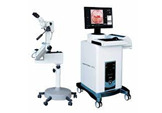	These devices are used inside hospitals.	Imaging devices with connectivity, such as X-rays and lab devices.

**Table 2:** The attack targeted devices and networks in IoMT.

Layer	Attacks	References
Device	Physical sensor/node tampering	[17]
	False data injection	[18,19]
	Resource depletion attacks (battery drain, sleep deprivation, buffer overflow)	[20]
	Side-channel	[7,21]
	Hardware Trojan	[22,23]
	Eavesdropping	[24]
	Ransomware	[25,26]
Network	Denial of Service (DoS) and distributed DoS (DDoS)	[24,27]
	Man in the middle (MIM)	[27]
	Eavesdropping attack	[24]
	Replay	[27,28]
	Botnet	[29]
	Jamming	[14]
	Flooding	[24,30]

**Table 3:** The average accuracy, CPU time, and memory usage of the incremental learning methods applied on the ToNT-IoT dataset.

Individual Sensor Dataset	Method	Average Accuracy (%)	Average Time (s)	Average Memory (MiB)
IoT-fridge	IKNN	99.72	8.96	2.8
	INB	99.93	3.16	1.80
	HTMC	99.42	**1.97**	**0.61**
	HTNB	99.92	2.08	**0.61**
	HTNBA	99.92	2.04	**0.61**
	WHTE	**99.95**	5.58	2.90
Garage door	IKNN	99.80	9.08	2.51
	INB	99.98	2.78	2.30
	HTMC	98.19	2.37	**0.71**
	HTNB	99.98	3.30	**0.71**
	HTNBA	99.96	**2.28**	**0.71**
	WHTE	**99.99**	8.68	3.99
GPS tracker	IKNN	99.79	9.32	2.46
	INB	99.34	**3.25**	2.53
	HTMC	90.34	32.32	**0.79**
	HTNB	99.73	42.13	**0.79**
	HTNBA	99.76	25.96	**0.79**
	WHTE	**99.80**	80.15	4.19
Modbus	IKNN	**100.00**	9.85	3.16
	INB	**100.00**	2.70	2.82
	HTMC	**100.00**	**0.67**	**0.48**
	HTNB	**100.00**	1.15	**0.48**
	HTNBA	**100.00**	1.05	**0.48**
	WHTE	**100.00**	3.74	3.87
Motion light	IKNN	99.80	10.59	3.04
	INB	98.42	**3.92**	3.23
	HTMC	96.55	13.60	**0.95**
	HTNB	96.55	13.55	**0.95**
	HTNBA	99.87	11.78	**0.95**
	WHTE	**99.90**	40.46	5.25
Thermostat	IKNN	99.90	8.38	3.00
	INB	**100.00**	2.96	2.94
	HTMC	**100.00**	**1.52**	**0.72**
	HTNB	**100.00**	2.05	**0.72**
	HTNBA	**100.00**	1.90	**0.72**
	WHTE	**100.00**	5.67	4.45
Weather sensor	IKNN	99.82	9.51	2.75
	INB	99.95	2.83	1.81
	HTMC	99.98	**1.24**	**0.54**
	HTNB	99.95	1.75	**0.54**
	HTNBA	99.97	1.58	**0.54**
	WHTE	**100.0**	5.83	3.68

**Table 4:** The ANOVA test results, where accuracy is the independent variable.

	Classifiers					
	IKNN	INB	HTMC	HTNB	HTNBA	WHTE
**Number of Datasets**	7	7	7	7	7	7
**Sum**	698.83	697.62	684.48	696.13	699.48	699.62
**Average**	99.83	99.66	97.78	99.45	99.93	99.95
**Details of Result**						
**Source**	*SS*	*df*	*MS*	*F*	*p*-value	
**Between classifiers**	24.08	5	4.82	2.00	0.10	
**Within classifiers**	86.52	36	2.40	-	-	
**Total**	110.61	41	-	-	-	

**Table 5:** The ANOVA test results, where computation time is the independent variable.

	Classifiers					
	IKNN	INB	HTMC	HTNB	HTNBA	WHTE
**Datasets #**	7	7	7	7	7	7
**Sum**	65.69	21.60	53.69	66.01	46.59	150.11
**Average**	9.38	3.09	7.67	9.43	6.66	21.44
**Details of Result**						
**Source**	*SS*	*df*	*MS*	*F*	*p*-value	
**Between classifiers**	1366.34	5	273.27	1.27	0.30	
**Within classifiers**	7743.23	36	215.09	-	-	
**Total**	9109.57	41	-	-	-	

**Table 6:** The ANOVA test results, where memory usage is the independent variable.

	Classifiers					
	IKNN	INB	HTMC	HTNB	HTNBA	WHTE
**Datasets #**	7	7	7	7	7	7
**Sum**	19.72	17.43	4.80	4.80	4.80	28.33
**Average**	2.82	2.49	0.69	0.69	0.69	4.05
**Details of Result**						
**Source**	*SS*	*df*	*MS*	*F*	*p*-value	
**Between classifiers**	71.56	5	14.31	88.33	2.5 × 10^−19^	
**Within classifiers**	5.83	36	0.16	-	-	
**Total**	77.39	41	-	-	-	

**Table 7:** The average accuracy, CPU time, and memory usage of the incremental learning methods applied on the NetFlow-ToNIoT dataset.

Dataset	Method	Average Accuracy (%)	Average Time (s)	Average Memory (MiB)	Average Precision (Normal)	Average Precision (Attack)	Average Recall (Normal)	Average Recall (Attack)
NetFlow-ToNIoT	IKNN	98.79	184.69	1.15	98.8	98.9	98.82	88.22
	INB	97.62	8.47	0.22	96.7	95.3	94.4	98.7
	HTMC	99.01	**3.90**	**0.08**	99.6	99.6	99.6	99.6
	HTNB	98.94	5.75	**0.08**	99.5	98.2	98.91	99.7
	HTNBA	99.01	5.02	**0.08**	99.7	99.7	98.99	99.1
	WHTE	**100.00**	12.89	0.37	**100.00**	**100.00**	**100.00**	**100.00**

**Table 8:** The comparison between the main results of this study and those reported in the literature.

Ref.	Domain	Architecture	Lightweight	Device Specs	Detection	Best Testing Accuracy (%)	Type/Name of Dataset	Classification Type	Type of Learning	Details on IoMT System and Fog Architecture	Complexity Metrics	Statistical Comparison	Splitting Method
[45]	IoMT	Cloud-Fog	No	CPU 2.20 GHz (10 cores, 13.75 MB L3 Cache), and 128 GB RAM	Network-based	96.35	Network packet/ToNIoT	Binary	Batch	No	Not considered	No	Holdout Train-test (80:20)
[44]	IoMT	Fog	No	Intel core i7 CPU processor and 16 GB RAM.	Network-based	98.19	Network packet/NSL-KDD	Binary	Batch	No	Not considered	No	Holdout Train-test (80:20)
[38]	Agriculture 4.0	Fog	No	Google Collaboratory supplied by GPU	Network-based	98.00	Network packet (CIC-DDoS2019 TON_IoT)	Multiclass	Batch	No	Not considered	No	Holdout Train-test (80:20)
[39]	IoT	Fog	No	Core (TM) i7- 6700 processor with 16 GB RAM	Network-based	93.44	Network packet (Bot-IoT)	Multiclass	Batch	No	Not considered	No	Holdout Train-test
[40]	IoT	Fog	No	-	Network-based	98.88	Network packet (Hogzilla Dataset)	Binary	Batch	No	Not considered	No	Holdout Train-test
This work	IoMT	Edge-Fog	Yes	CPUs ≈ 2.2 GHz (4 cores, 3 MB L3 Cache), and 8 GB RAM	Hybrid (Host and Network-based)	100.00	NetFlow and Sensors datasets (ToNIoT sensors and NetFlowToNIoT)	Multiclass for sensors and binary for NetFlow	Incremental	yes	Considered	Yes	Windowing (test-train)
